# Reviewer acknowledgement 2013

**DOI:** 10.1186/1471-2202-15-27

**Published:** 2014-03-18

**Authors:** Tim Shipley

**Affiliations:** 1 BioMed Central, Floor 6, 236 Gray's Inn Road, London WC1X 8HB, UK

## Abstract

**Contributing reviewers:**

The editors of BMC Neuroscience would like to thank all our reviewers who have contributed to the journal in Volume 13 (2013).

## 

Stefanie Abel

Germany

Roger Adan

Netherlands

Fabienne Agasse

United States of America

Espana Agnès

France

Alexander Agoulnik

United States of America

Tariq Ahmed

Belgium

Zaghloul Ahmed

United States of America

Zubair Ahmed

United Kingdom

Michael Akins

United States of America

Haruhiko Akiyama

Japan

Noor Alam

United States of America

Roger Albin

United States of America

Alessio Alfieri

United Kingdom

Mark Alkema

United States of America

Shelley Allen

United Kingdom

Manuel Alvarez Dolado

Spain

Julià L Amengual

Spain

Miroslava Anderova

Czech Republic

Balasubramaniam Annamalai

United States of America

Andrea Antal

Germany

Lawrence Appelbaum

United States of America

Juan Carlos Arevalo

Spain

Ubaldo Armato

Italy

William Armstead

United States of America

Rebecca Ashare

United States of America

Mariana Astiz

Spain

Denize Atan

United Kingdom

Coleen Atkins

United States of America

N. Ahmad Aziz

Netherlands

Sergio Bagnato

Italy

Claudia Bagni

Belgium

Joerg Bahlmann

Germany

Travis Baker

Canada

Nigel Bamford

United States of America

Andrea Banfi

Switzerland

Jianxin Bao

United States of America

Clive Bate

United Kingdom

Giorgio Stefano Battaglia

Italy

Tobias Bäumer

Germany

Steven L Bealer

United States of America

Marie Hoeger Bement

United States of America

Alexandra Bendixen

Germany

Peter Bergold

United States of America

David Berron

Germany

Yasumasa Bessho

Japan

Matthew Betts

Germany

Frederike Beyer

Germany

Ferdinand Binkofski

Germany

David Biron

United States of America

Brenda Bloodgood

United States of America

Matteo Bologna

Italy

Ubaldo Bonuccelli

Italy

Mei Ying Boon

Australia

Stephanie Booth

Canada

Cesar Borlongan

United States of America

Matthew Boyko

Israel

Angela Brennan

United States of America

Gregory Brewer

United States of America

Holly Bridge

United Kingdom

Filippo Brighina

Italy

Guy Brock

United States of America

Emmanuel Brouillet

France

Rachel Brown

Canada

Stefan Brudzynski

Canada

Norbert Bruggemann

Denmark

Susanne Brummelte

Canada

Aneta Brzezicka

Poland

Luigi Bubacco

Italy

Eva C Bunk

Germany

Nico Bunzeck

Germany

Josh Burk

United States of America

Laura Calza

Italy

Gabriela Cantarero

United States of America

Filippo Caraci

Italy

Caty Casas

Spain

Nathan Cashdollar

Italy

Andrea Eugenio Cavanna

United Kingdom

Maximilien Chaumon

France

L. Leon Chen

United States of America

Lu Chen

United States of America

Michael Chen

United States of America

Sidi Chen

United States of America

Irene Cheng

Taiwan

Yu-Chin Chiu

United States of America

Eric Cho

Hong Kong

Vivian Ciaramitaro

United States of America

Norberto Coimbra

Brazil

Boaz Cook

United States of America

Laura Corns

United Kingdom

Valerie Coronas

France

Daniel N. Cox

United States of America

Bernard Crespi

Canada

Adolfo Cuadra

United States of America

Carsten Culmsee

Germany

Gennady Cymbalyuk

United States of America

Ashraf Dahaba

Austria

Christina Dalla

Greece

Mark Dallas

United Kingdom

Vladimer Darsalia

Sweden

Venkata Ramesh Dasari

United States of America

Fred Davis

United States of America

Alain de Cheveigné

France

Elvira De Leonibus

Italy

Maarten De Vos

Germany

Nicole Déglon

Switzerland

Alberto Del Arco

Spain

Maria A. Deli

Hungary

Igor Delvendahl

Germany

Thomas DeMarse

United States of America

Catherine Dermon

Greece

Krishnan Dhandapani

United States of America

Rudi D'Hooge

Belgium

James Dillman

United States of America

Maria José Diógenes

Portugal

Ulrich Dirnagl

Germany

Nuria Donamayor Alonso

Germany

Vonetta Dotson

United States of America

Robert Dowman

United States of America

Carlos Duarte

Portugal

Celia Duarte Cruz

Portugal

Michael Dulas

United States of America

Matthias Dümpelmann

Germany

John Duncan

United Kingdom

Luc Dupuis

France

Patrick Dutar

France

Alfred Effenberg

Germany

Olga Efimova

Russian Federation

Hannelore Ehrenreich

Germany

Ingrid Ehrlich

Germany

Piet Eikelenboom

Netherlands

Ana Elgoyhen

Argentina

Dan-Mikael Ellingsen

Sweden

Eskil Elmér

Sweden

Takeshi Enomoto

Japan

Peter Enticott

Australia

Michael Falkenstein

Germany

Zhou Fei

China

Dirk Feldmeyer

Germany

Pedro Fernández-Llebrez

Spain

Merari Ferrari

Brazil

Donna Ferriero

United States of America

Chase Figley

United States of America

Bonnie Firestein

United States of America

Dan Foti

United States of America

Laura Frago

Spain

Graham Fraser

Canada

Lluis Fuentemilla

United Kingdom

Inna Gaisler-Salomon

Israel

Maria Joao Gama

Portugal

Subramaniam Ganesh

India

Justine Gatt

Australia

David Gaze

United Kingdom

Eldon Geisert

United States of America

Ana Genaro

Argentina

Alain Ghysen

France

Claire Gibson

United Kingdom

Carsten Giessing

Germany

Stephen Glatt

United States of America

Sebastian Gluth

Germany

Ilan Gobius

Australia

Martin Goettlich

Germany

Carlos Gómez

Spain

Guillermo Gonzalez-Burgos

United States of America

Paul Gordon

United States of America

Daniel Graf

Greece

Jorg Grandl

United States of America

William Green

United States of America

William Griffith

United States of America

Laurent Groc

France

Sergiu Groppa

Germany

Agnès Gruart

Spain

Marcus Grueschow

Switzerland

Paolo Gubellini

France

Nicolas Guerout

France

Jun Guo

China

Kurt Haas

Canada

Hashem Haghdoost-Yazdi

Iran

Matthew Hale

Australia

Edward Hall

United States of America

Javad Hami

Iran

Kurt Hammerschmidt

Germany

Bill Hansson

Germany

Mitsuhiro Hashimoto

Japan

Sadayuki Hashioka

Canada

Silke Haverkamp

Germany

Xiaolong He

Venezuela

Ying He

United States of America

Stephan Heermann

Germany

Marcus Heldmann

Germany

Craig Henriquez

United States of America

Denis Herve

France

Johannes Hewig

Germany

Johanne Higgins

Canada

Rachel Hill

Australia

Kate Himmelmann

Sweden

Arlene Hirano

United States of America

Helena Hogberg

United States of America

Wugang Hou

China

Steven Hsiao

United States of America

Bo Hua Hu

United States of America

Xun Huang

China

Ying-Zu Huang

Taiwan

Sandrine Humez

France

Fatima Husain

United States of America

Rene Huster

Germany

Alan Hutson

United States of America

Jeffrey Iliff

United States of America

Nibaldo Inestrosa

Chile

Masako Isokawa

United States of America

Koichi Iwata

Japan

Corentin Jacques

Belgium

Lyn Jakeman

United States of America

Natalia Jaworska

Canada

Glen Jeffery

United Kingdom

Nick Jeffery

United States of America

Jaeseung Jeong

Korea, South

Suresh Jesuthasan

Singapore

Zhengping Jia

Canada

Haitao Jiang

China

Dianna Johnson

United States of America

Erin Johnson Venkatesh

United States of America

Jamie Johnston

Canada

Won-Kyu Ju

United States of America

Nikolai Jung

Germany

Abigail Kalmbach

United States of America

Masahiro Kameda

Japan

Rafal Kaminski

Belgium

Wessel Karl

Germany

Don Katz

United States of America

Charanjit Kaur

Singapore

Hiroshi Kawasaki

Japan

Wolfram Kawohl

Switzerland

Alex Keene

United States of America

David Kelly

United Kingdom

Mushfiquddin Khan

United States of America

June Sic Kim

Korea, South

Yongsung Kim

United States of America

Adam King

United States of America

Jochen Klucken

Germany

Maria Knikou

United States of America

Christina Knuepffer

Australia

Noriko Koganezawa

Japan

Jari Koistinaho

Finland

Ulla Kopp

United States of America

Tomasz Kordula

United States of America

Peter Koulen

United States of America

Dimitrios Kourtis

Belgium

Neil Kowall

United States of America

Ulrike Krämer

Germany

Leszek Kubin

United States of America

Kin-ya Kubo

Japan

Stefanie Kuerten

Germany

Irina Kufareva

United States of America

Ron Kupers

Denmark

Kumi Kuroda

Japan

John Kwok

Australia

Charalambos Kyriacou

United Kingdom

Peter Lackner

Austria

Pauline Lafenetre

France

Bing Lang

United Kingdom

Mark LeDoux

United States of America

Chia-lin Lee

Taiwan

Hwee Ling Lee

Germany

Michael Lee

United Kingdom

Seung-Hwan Lee

Korea, South

Jose Lemos

United States of America

Damiana Leo

Italy

Christopher Leonardo

United States of America

Noelle L'Etoile

United States of America

L Stan Leung

Canada

Jan Lewerenz

Germany

Andrew Leynes

United States of America

Bin Li

United States of America

Qingli Li

China

Weiquan Li

United States of America

Xin Li

United States of America

Yonggang Li

United States of America

Shih-Chieh Lin

United States of America

Dan Lindholm

Finland

Eng-Ang Ling

Singapore

Ralf Linker

Germany

Hui-li Liu

China

Doug Lobner

United States of America

Mary Kay Lobo

United States of America

David Lodge

United Kingdom

Martin Lotze

Germany

Andreas Löw

Germany

Diego Lozano-Soldevilla

Netherlands

Mei Lu

United States of America

Thomas Lukas

United States of America

Wenyu Luo

United States of America

Jinglei Lv

China

Heidi Muller

Denmark

Daqing Ma

United Kingdom

Weiya Ma

Canada

Wendy Macklin

United States of America

Kathy Magnusson

United States of America

Malek Makki

Switzerland

Francesca Mangialasche

Sweden

Roger Marek

Australia

Valeria Marigo

Italy

Stephen Martin

United Kingdom

Sergio Martinoia

Israel

Valery Matarazzo

France

Chantal Mathis

France

Gregory Matuszek

United States of America

Philip McGoldrick

United Kingdom

Christopher McNorgan

Canada

Magdalena Mendez-Lopez

Spain

Ahmet Mentese

Turkey

Nicola Mercuri

Italy

Philipp Mergenthaler

Germany

Charles Meshul

United States of America

Robert Miller

United States of America

Mariarosaria Miloso

Italy

Berge Minassian

Canada

Hiroyuki Mino

Japan

Jyoti Mishra

United States of America

Tsuyoshi Miyakawa

Japan

Toshiyuki Mizui

Japan

Hiren Modi

United States of America

Bahram Mohammadi

Germany

Raffaella Molteni

Italy

Paola Morales

Chile

Stephan Moratti

Spain

Nadia Mores

Italy

Yasuo Mori

Japan

Andrea Mothe

Canada

Khyobeni Mozhui

United States of America

Yukitoshi Nagahara

Japan

Shinsuke Nakagawa

Japan

Takayuki Nakagawa

Japan

Esperanza Navarro Pardo

Spain

Phoebe Neo

New Zealand

Irene Neuner

Germany

Zhen Ni

Canada

Natalia Ninkina

United Kingdom

Michael Nitsche

Germany

Matthias Nitschke

Germany

Margaret Niznikiewicz

United States of America

Mark Obermann

Germany

John O'Connor

Ireland

Sharon ONeill

United States of America

Wei-Yi Ong

Singapore

Juan Orellana

Chile

Brandi Ormerod

United States of America

Roman Osinsky

Germany

Yasuomi Ouchi

Japan

Hiroshi Oue

Japan

Satu Pakarinen

Finland

Dinesh Pal

United States of America

Michele Papa

Italy

Vinay Parikh

United States of America

Thomas Park

United States of America

Juana Maria Pasquini

Argentina

Jesús Pastor

Spain

Samirkumar Patel

United States of America

William Pearce

United States of America

Keith Pennypacker

United States of America

Santiago Perez-Lloret

France

Muriel Perron

France

Armando Perrotta

Italy

Rasmus Petersen

United Kingdom

Linda Phillips

United States of America

Andrzej Pilc

Poland

Alberto Pisoni

Italy

Konstanze Plaschke

Germany

Markus Ploner

Germany

Ulrich Pomper

Germany

Douglas Portman

United States of America

Gro Povlsen

Denmark

Ashley Pringle

United Kingdom

Andreas Prokop

United Kingdom

Shuhong Qiao

United States of America

Angelo Quartarone

Italy

Eugenia Radulescu

United Kingdom

Chander Raman

United States of America

Vickram Ramkumar

United States of America

Tomi Rantamäki

Finland

Yehoash Raphael

United States of America

Frank Rattay

Austria

John Redell

United States of America

Damian Refojo

Germany

Owen Rennert

United States of America

Sylvia Richter

Austria

Larry Roberts

Canada

George robertson

Canada

Borja Rodríguez

Spain

Antoni Rodriguez-Fornells

Spain

Brigitte Roeder

Germany

Ardi Roelofs

Netherlands

Patricia Rojas

Mexico

Anne-Sophie Rolland

United States of America

Mohammad Ronaghi

United States of America

Merri Rosen

United States of America

Susanna Rosi

United States of America

Bruno Rossion

Belgium

Panos Roussos

United States of America

Malgorzata Rozanowska

United Kingdom

Jascha Ruesseler

Germany

Philipp Ruhnau

Italy

Ruth Ruscheweyh

Germany

Jane Rylett

Canada

Mariusz Sacharczuk

Poland

Harutoshi Sakakima

Japan

Shuzo Sakata

United Kingdom

Katrin Sakreida

Germany

Takeshi Sakurai

Japan

Maria Victoria Sánchez-Gómez

Spain

Ursula Sandau

United States of America

David Sanderson

United Kingdom

Terence Sanger

United States of America

Pascual Sanz

Spain

Lieven Schenk

Germany

Roland Schoenherr

Germany

Menno Schoonheim

Netherlands

Derek Schreihofer

United States of America

Markus Schubert

Germany

David Schulz

United States of America

Jeremy Seamans

Canada

Thomas Shea

United States of America

Haroon Sheikh

Canada

Koji Shibasaki

Japan

Feng-Shiun Shie

Taiwan

Marshall Shuler

United States of America

Sandy Shultz

Australia

Donald Simone

United States of America

Shiva Singh

Canada

Indrani Sinha-Hikim

United States of America

Dario Siniscalco

Italy

John Slevin

United States of America

Deanna Smith

United States of America

Janette Smith

Australia

Robert Smith

United States of America

Jean-Jacques Soghomonian

United States of America

Farida Sohrabji

United States of America

Maria Angela Sortino

Italy

Marcia Sosthenes

Brazil

João Sousa

Portugal

Ulla Sovio

United Kingdom

Sarah Spiegel

United States of America

Laure Spieser

France

Kartik Sreenivasan

United States of America

James St John

Australia

Wolfgang Stein

Germany

Jena Steinle

United States of America

Christian Stoppel

Germany

Judith Strong

United States of America

Joerg Strotmann

Germany

Antonio Suppa

Italy

Matthew Sutherland

United States of America

Masatoshi Suzuki

United States of America

Clive Svendsen

United States of America

Anand Swaroop

United States of America

Gregor R. Szycik

Germany

Ameer Taha

United States of America

Saeid Taheri

United States of America

Hideaki Tanaka

Japan

Victor Tapias

United States of America

Somporn Techangamsuwan

Thailand

Alana M Thackray

United Kingdom

Thomas Theil

United Kingdom

Elizabeth Thomas

United States of America

Veronika Thorns

Germany

Michael Tobia

Germany

Takuro Tojima

Japan

Lars Tonges

Germany

Maggie Toplak

Canada

Peter Trillenberg

Germany

Regina Trollmann

Germany

Matt Trudeau

United States of America

Nelson Trujillo-Barreto

Cuba

Yiu-Kei Tsang

Hong Kong

Kwong-Chung Tung

Taiwan

Toos van Beijsterveldt

Netherlands

Frank van Bel

Netherlands

Lise van der Haegen

Belgium

Bart van der Worp

Netherlands

Michiel van Elk

Switzerland

Arend van Gemmert

United States of America

Geerten P. van Nieuw Amerongen

Netherlands

Cindy van Velthoven

Netherlands

Akshya Vasudev

Canada

Alessandro Vercelli

Italy

Kata Orsolya Veres-Nyéki

Hungary

Michael Villiger

Switzerland

Scott Waddell

United Kingdom

Chengzhong Wang

United States of America

Ling Wei

United States of America

Norbert Weidner

Germany

Fletcher White

United States of America

Holger Wiese

Germany

Roland Wiest

Switzerland

Kim Wilkinson

United States of America

Daniel Wiswede

Germany

George Wittenberg

United States of America

Martin Woon

United States of America

Lisa Wright

Canada

Han-Ping Wu

Taiwan

Jian-young Wu

United States of America

Kit Wu

United Kingdom

Christopher Wyatt

United States of America

Yang XIang

United States of America

Ye Xiong

United States of America

Bainan Xu

China

Fuqiang Xu

China

Shawn Xu

United States of America

Ipek Yalcin

France

Kazuhiko Yamaguchi

Japan

Nobuhiko Yamamoto

Japan

Takamitsu Yamamoto

Japan

Chae Ha Yang

Korea, South

Hao Yang

China

Li Yang

United States of America

Shaohua Yang

United States of America

Anatoliy Yashin

United States of America

Hiroshi Yatsushige

Japan

Ping Ye

United States of America

Zheng Ye

United Kingdom

Zheng Ye

Germany

Shin-Young Yim

Korea, South

Hao-Jun You

China

Zhanyang Yu

United States of America

Ting Yuan

China

Bing Zang

United States of America

Richard Zeman

United States of America

Karen Zentgraf

Germany

Chenggang Zhang

China

Jie Zhang

United States of America

John Zhang

United States of America

Wei Zhang

United States of America

Yumin Zhang

United States of America

Yun Zhang

United States of America

Chunjie Zhao

China

Yanxiang Zhao

Hong Kong

Zhangwu Zhao

China

Cui Qing Zhu

China

Dong-Ya Zhu

China

Lei Zhu

United States of America

Jian Zou

China

